# Brazilian experts' consensus on the treatment of infantile epileptic spasm syndrome in infants

**DOI:** 10.1055/s-0043-1772835

**Published:** 2023-10-04

**Authors:** Letícia Pereira de Brito Sampaio, Adélia Maria de Miranda Henriques-Souza, Mariana Ribeiro Marcondes da Silveira, Lisiane Seguti, Mara Lúcia Schmitz Ferreira Santos, Maria Augusta Montenegro, Sérgio Antoniuk, Maria Luíza Giraldes de Manreza

**Affiliations:** 1 Universidade de São Paulo, Faculdade de Medicina, Hospital das Clínicas, São Paulo SP, Brazil.; 2 Instituto de Medicina Integral Professor Fernando Figueira, Recife PE, Brazil.; 3 Hospital da Restauração, Recife, PE, Brazil.; 4 Universidade de Brasília, Faculdade de Medicina, Área da Medicina da Criança e do Adolescente, Brasília DF, Brazil.; 5 Hospital Pequeno Príncipe, Departamento de Neurologia Pediátrica, Curitiba PR, Brazil.; 6 University of California San Diego, Rady Children's Hospital, San Diego CA, United States.; 7 Universidade Federal do Paraná, Departamento de Pediatria, Curitiba PR, Brazil.

**Keywords:** Spasms, Infantile, Treatment, Epilepsy, Espasmos Infantis, Tratamento, Epilepsia

## Abstract

**Background** Infantile epileptic spasms syndrome (IESS) is a rare but severe condition affecting children early and is usually secondary to an identifiable brain disorder. It is related to psychomotor deterioration in childhood and epilepsy in adult life. Treatment is challenging as infantile spasms may not respond to most antiseizure medication, and relapse is frequent.

**Objective** To evaluate the literature regarding treatment of IESS and provide a practical guidance to a healthcare system with limited resources.

**Methods** An expert committee from the Brazilian Society of Child Neurology reviewed and discussed relevant scientific evidence in the treatment of IESS regarding the drugs available in Brazil.

**Results** Oral prednisolone and vigabatrin are the most common drugs used as first-line therapy; they are efficient and affordable therapy as both are available in the Brazilian unified health system (SUS, in the Portuguese acronym). Intramuscular adrenocorticotropic hormone (ACTH) presents similar efficacy as oral prednisolone but has a higher cost and is not available in Brazil. Other antiseizure medications such as topiramate, levetiracetam, or benzodiazepines have limited response and are prescribed as adjuvant therapy. If the health service has nutritionists, a ketogenic diet should be implemented for those not responding to hormonal and vigabatrin treatment. Epilepsy surgery is mainly indicated for patients with focal lesions that do not respond to pharmacological therapy.

**Conclusion** Early treatment of IESS with efficient drugs is feasible in our country. Using standard protocols increases the odds of achieving complete cessation in a shorter time and decreases relapse.

## Resumo

**Antecedentes** A síndrome do espasmo epiléptico infantil (IESS) é uma condição rara, mas grave, que afeta crianças precocemente e geralmente é secundária a um distúrbio cerebral identificável, estando relacionada a deterioração psicomotora na infância e a epilepsia na vida adulta. O tratamento é desafiador, pois os espasmos infantis podem não responder à maioria dos medicamentos anticrises e as recidivas são frequentes.

**Objetivo** Avaliar a literatura sobre o tratamento de IESS e fornecer uma orientação prática para um sistema de saúde com recursos limitados.

**Métodos** Um comitê de especialistas da Sociedade Brasileira de Neurologia Infantil revisou e discutiu evidências científicas relevantes no tratamento da IESS em relação aos medicamentos disponíveis no Brasil.

**Resultados** Prednisolona oral e vigabatrina são os fármacos mais comumente usados como terapia de primeira linha; são eficientes e acessíveis, já que ambos estão disponíveis no sistema único de saúde brasileiro (SUS). O ACTH intramuscular apresenta eficácia semelhante à prednisolona oral, mas tem custo mais elevado e não está disponível no Brasil. Outros medicamentos anticonvulsivos, como topiramato, levetiracetam ou benzodiazepínicos, têm resposta limitada e são prescritos como terapia adjuvante. Se o serviço de saúde tiver nutricionista, deve-se implementar dieta cetogênica para aqueles que não respondem ao tratamento hormonal e vigabatrina. A cirurgia de epilepsia é indicada principalmente para pacientes com lesões focais que não respondem à terapia farmacológica.

**Conclusão** O tratamento precoce da IESS com fármacos eficazes é factível em nosso meio. O uso de protocolos padronizados aumenta as chances de alcançar a cessação completa em um tempo menor e diminui a recaída.

## INTRODUCTION

Infantile spasm is a type of seizure occurring in children < 2 years old characterized by clinical spasms with sudden flexor, extensor, or mixed flexor-extensor symmetrical contractions of the head, neck, trunk, and limbs, occurring in clusters, lasting 1 to 2 seconds, usually but not necessarily accompanied by hypsarrhythmia. 1 2 West syndrome (WS), frequently considered a synonym for infantile spasms, is reserved for clinical spasms in clusters with hypsarrhythmia on an electroencephalogram (EEG). 1 According to the new classification proposed by the International League Against Infantile Epilepsy, infantile epileptic spasm syndrome (IESS) includes infants with WS and with epileptic spasms who do not fulfill all the criteria for WS. 3 Developmental delay before the onset of spasms is no longer necessary for diagnosis since some children, especially those with unknown etiology, may have subtle changes, such as reduced social smile alone. 1 Therefore, treatment should not be delayed in cases where the developmental alteration is mild.

Infantile epileptic spasm syndrome is the most common epileptic encephalopathy affecting children in the 1 ^st^ year of life, 4 with an estimated incidence of 2.9 to 4.5 per 10,000 live births. 5 6 7 More than 50% of patients have an identifiable underlining disorder, such as brain malformations, hypoxic-ischemic encephalopathy, tuberous sclerosis complex (TSC), Down syndrome, and other genetic disorders. 8

The treatment of IESS is challenging, and most patients develop pharmacoresistant epilepsy, cognitive impairment, and autism spectrum disorder. 2 9

Early intervention is one of the most important factors in preventing an unfavorable outcome, and the therapeutic choice must consider access to medical care and costs, especially in low to middle-income countries. In the present paper, we aimed to provide practical guidance in treating IESS in Brazil, considering the limitations of our healthcare system.

## METHODS

The Brazilian Society of Child Neurology composed an expert committed ( *n* = 9) with extensive experience in epilepsy and familiar with the challenges of the Brazilian unified healthcare system (SUS, in the Portuguese acronym).

**Figure 1:**
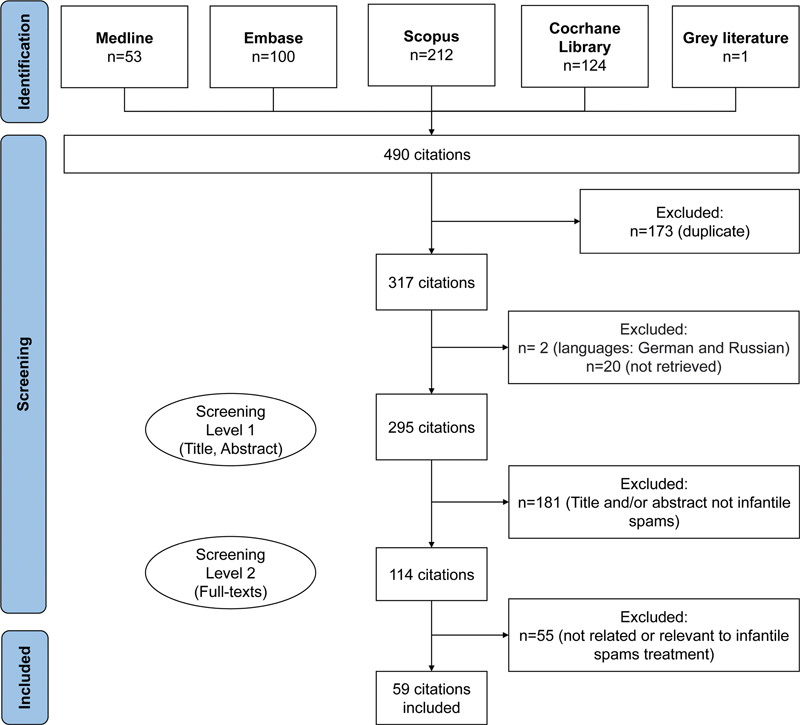
Flowchart of the literature search.

A systematic review was performed between April and May 2021 using the Medline (PubMed version), EMBASE (Ovid), SCOPUS, and Cochrane Library databases, with the following search terms: *infantile spasm* , *west syndrome* , *triad: infantile spasms, hypsarrhythmia and mental deficiency/intellectual disability/mental retardation* , including *treatment* and *children* . The same terms were used for research in the gray literature: Brazilian Infantile Neurology Society, Brazilian Academy of Neurology, Brazilian League of Epilepsy, Brazilian Pediatric Society. The inclusion criteria were meta-analyses of randomized and nonrandomized clinical trials or observational studies, double-blind or open randomized clinical trials, observational studies, and case reports published in the past 20 years, in English or Portuguese. Preclinical studies were excluded. The flowchart of the literature search is illustrated in Figure 1 .

**Figure 2:**
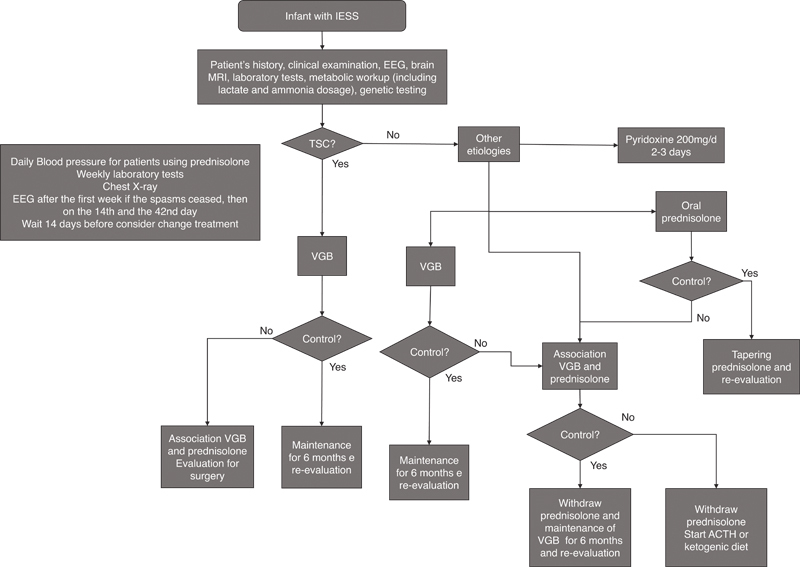
Infantile spasms management algorithm.

The experts received the results of the literature research and a questionnaire to be completed individually and anonymously. The group established the most appropriate approach in treating IESS in Brazil, considering the limitations of the healthcare system in this country. An algorithm was created to help guide clinical decision-making ( Figure 2 ).

## FIRST-LINE THERAPY

Large randomized controlled trials (RCT) support oral prednisolone or intramuscular adrenocorticotropic hormone (ACTH) as the first-line therapy for IESS. Vigabatrin (VGB) is also an option in the first-line treatment of IESS.

### Hormonal therapy: oral corticosteroids versus intramuscular ACTH

Oral prednisolone may show a faster response than intramuscular ACTH, but no difference in long-term efficiency has been observed between treatments.

The UKISS study 10 was an RCT including 107 children with IESS, randomized in hormonal treatment (oral prednisolone 40 to 60 mg/day or intramuscular synthetic ACTH, 0.5 mg up to 0.75 mg every other day) versus VGB (minimum of 120 mg/kg/day and maximum of 150 mg/kg/day). Seventy percent of patients in the prednisolone group and 76% in the ACTH group achieved complete cessation of spasms in 14 days ( *p* = 0.61). 10

A single-blind RCT 11 evaluated 92 children with WS, receiving either 40 to 60 mg/day of oral prednisolone or 40 to 60 IU (0.5 to 0.75 mg) every other day of intramuscular synthetic ACTH. After 14 days of treatment, prednisolone showed higher improvement of the hypsarrhythmia severity score than ACTH (7.95 ± 2.76 versus 6.00 ± 2.61, respectively, *p* < 0.01). 11 In 58.3% of children treated with prednisolone, spasms ceased completed versus 36.7% with ACTH ( *p* = 0.03). 12 Time to remission was shorter with prednisolone, with a mean of 3.85 ± 2.4 days versus 8.65 ± 3.7 days ( *p* = 0.001). 12 After 28 days, 31.2% of the prednisolone group remained without spasms versus 12.2% of the ACTH group ( *p* = 0.008). 12 After 6 months ( *n* = 82), 58.3% of the patients treated with prednisolone were spasms-free versus 44.9% of those treated with ACTH ( *p* = 0.19). 13 After 4 years, among the 65 children who were re-evaluated, 57% continued to have seizures, and 18.5% continued having epileptic spasms, with no difference between children initially treated with oral prednisolone or ACTH (p > 0.05). 14

In an RCT 15 ( *n* = 34) evaluating intramuscular ACTH (100 UI/m ^2^ /day) versus oral prednisolone (4 mg/kg/day) for 2 weeks, no difference was observed in the cessation of spasms or in the time to achieve cessation.

In a retrospective evaluation of synthetic ACTH (tetracosactide) versus prednisolone (2–3 mg/kg/day divided into 2 doses) in the treatment of IESS including 105 children, 82% of the ACTH group had ≥ 50% reduction in seizure frequency during treatment, and 33% remained seizure-free 6 six weeks. 16 A total of 71% of the prednisolone group presented a decrease in seizure frequency during treatment, and 23% after the end of treatment. 16 The authors did not observe any difference between groups ( *p* = 0.41). 16

A meta-analysis from the UKISS and ICISS study 17 confirmed that both prednisolone and ACTH are efficient as initial monotherapy in children with IESS, with no significant difference between them (treatment difference 7.8%, 95% confidence interval [CI]: - 8.7–24.3%; *p* = 0.34). Another meta-analysis included 5 RCTs ( *n* = 239) that compared oral corticosteroids and intramuscular synthetic ACTH. 18 The overall cessation of spasms was similar between treatments (odds ratio [OR)]: 0.54; 95%CI: 0.16–1.81; *p* = 0.32). 18 Neither high-dose prednisolone (4 mg/kg/day, maximum 60 mg/day) (OR: 1.01; 95%CI: 0.40–2.98; *p* = 0.87), nor low-dose (2 mg/kg/day) (OR: 0.13; 95%CI: 0.01–2.00; *p* = 0.14) showed any difference. 18 Resolution of hypsarrhythmia was also similar between groups (OR: 0.5; 95%CI: 0.12–2.13; *p* = 0.35), as well as the relapse rate (OR: 0.68; 95%CI: 0.19–2.40; *p* = 0.55), or subsequent epilepsy (OR: 0.84; 95%CI: 0.30–2.32; *p* = 0.73). 18 Concerning adverse events, no difference was observed in the incidence of hypertension, irritability, or infection. 18

In a retrospective chart review, 19 27 children with IESS received high-dose oral prednisolone (8 mg/kg/day, maximum 60 mg/day) and intramuscular ACTH (150 IU/m ^2^ /day) for nonresponders. A total of 63% of patients responded to oral prednisolone. 19 Among the remaining nonresponders, 40% presented complete remission of spasms with ACTH. 19 The relapse rate was 11.7% among prednisolone responders and 50% among ACTH responders. 19

### Dose and presentation of hormonal therapy

When using corticosteroids, clinicians should prescribe a high dose of oral prednisolone. In an open-label RCT comparing a high dose of prednisolone (4 mg/kg/day) versus the usual dose (2 mg/kg/day) in 63 children with IESS, 20 51.6% of the children treated with a high dose achieved complete cessation of seizures for at least 48 hours on day 14, versus 25% treated with the usual dose ( *p* = 0.03). The incidence of adverse events was similar between treatment groups. 20

In a retrospective evaluation, 21 87 children with IESS were treated with high-dose oral prednisolone, starting at 40 mg/day using the UKISS protocol, increased to 60 mg/day in case of nonresponse within 1 week. A total of 71.3% of patients were responders (complete cessation of spasms) after 2 weeks of treatment, and 64.4% had resolution of spasms and hypsarrhythmia. 21

In an RCT, 22 32 children with IESS were evaluated receiving either low (0.1 mg) or high (0.25 mg) intramuscular ACTH dose once a day for 1 week, then weekly decreasing to once every other day, twice a week, once a week, and once every 2 weeks until 8 weeks of treatment. Both treatment groups showed a reduction in seizures: 43% of the low-dose group and 56% of the high-dose group; however, patients treated with high doses had a higher incidence of agitation, sleep disorders ( *p* = 0.002), and Cushing syndrome ( *p* = 0.04). 22 Moreover, a retrospective cohort study 23 showed no difference between low-moderate (40 IU/day) versus high-dose (120 IU/day) natural ACTH, and natural versus synthetic ACTH, after 2 to 3 weeks of treatment.

Oral prednisolone should be the choice of treatment with corticosteroids, as oral treatment has lower costs and risks of adverse effects than intravenous administration. An open-label RCT 24 compared intravenous methylprednisolone with oral prednisolone to treat 60 patients with WS. This study showed similar results between groups, with complete remission at D14: 54.8% with intravenous versus 68.9% with oral ( *p* = 0.26). Intravenous methylprednisolone showed a faster response, with a mean of 5.4 ± 0.9 days versus 9.5 ± 2.6 days with oral prednisolone ( *p* < 0.0001). 24 Relapse occurred in 19.4% of patients receiving intravenous methylprednisolone in 6 weeks, but none in the oral prednisolone group (OR: 0.08; 95%CI: 0.004–1.5; *p* = 0.0242). 24

A retrospective study 25 included 28 children with IESS and assessed the efficacy of intramuscular ACTH versus dexamethasone in pulse therapy. Patients received synthetic intramuscular ACTH, starting at 15 to 20 IU/day, increasing to 20 IU/day every 2 weeks, until 120 IU/day for nonresponders, and then decreasing 20 IU/day every 2 weeks. 25 The pulse-therapy cycle comprised 20 mg/m ^2^ of intravenous dexamethasone/day for 3 days, an interval of 4 weeks per cycle, and a total of 5 cycles. 25 Treatments showed similar results, whereas 64.2% of the patients treated with ACTH were seizure-free versus 57.1% with pulse therapy. 25

### Timing for treatment

Treatment should not be delayed. Randomized trials showed the significant impact of a prolonged time between the onset of spasms and the onset of treatment (the lead time to treatment) in neurodevelopment. 26 Seventy-seven 4-year-old children from the UKISS study had a decrease of 3.9 points in the developmental score with the increase in lead time. 26 Children from the ICISS study with lead time to treatment > 2 months had also a greater developmental decline ( *p* = 0.0138). 27 This decision should be based on the aforementioned studies or higher evidence, if available, because observational studies could not reproduce the same results.

A retrospective chart review study, 28 evaluating the best timing to begin ACTH ( *n* = 90), showed no difference in cessation of spasm if treatment started within 1 month of the onset of seizures or afterwards (risk ratio [RR]: 1.00; 95%CI: 0.79–1.27; *p* = 1.00 for the 2-month evaluation; RR: 0.96; 95%CI: 0.74–1.24; *p* = 0.74 for the 6-month evaluation; RR: 1.00; 95%CI: 0.75–1.33; *p* = 1.00 for the 1-year evaluation).

Adrenocorticotropic hormone should be discontinued if patients do not achieve complete remission within 2 weeks of treatment. In a retrospective chart review of 42 patients treated with ACTH, 54% responded to treatment, and the mean time to complete response was 5.8 days (1 to 20 days), in which 96% responded in 2 weeks after initiation of treatment and 100% within 3 weeks. 29

### Vigabatrin

Vigabatrin is an antiseizure medication registered in Brazil but with limited availability in this country, especially in public services. Evidence correlates VGB with retinopathy and MRI structural abnormalities with extrapyramidal effects. 30 31 32 33 According to a systematic review 30 including 1,678 patients treated with VGB and 406 controls, the relative risk of VGB-associated visual field loss is 4.0 (95%CI: 2.9–5.5). The retinopathy may be related to the increase of gama-aminobutyric acid (GABA) and current scientific evidence on the effectiveness of taurine in preventing GBV retinopathy is limited and there is no definitive consensus. The findings in the VGB-associated brain abnormalities on MRI (VABAM) include diffusion restriction in the globus pallidus, the corpus callosum, the thalamus, the cerebellar dentate nuclei, the midbrain, and the brainstem; some patients are asymptomatic (more common), and others develop dyskinesias or acute encephalopathy. 31 Vigabatrin associated with ACTH increases the risk of fulminant VABAM, especially using high doses of ACTH. 31 Symptoms are reversible after the reduction or discontinuation of the drug, but there are reports of fatal encephalopathy. 31 32 33

The RCTs evaluating VGB are described in the next session of the present paper, for they are head-to-head trials versus hormonal treatment. Open-label and observational studies demonstrate the efficacy of VGB in treating children with IESS. In an open-label study, 34 clinicians treated 5 children with WS with VGB (starting with 5 to 10 mg/kg twice a day, up to 80 to 100 mg/kg in 10 days) in association with topiramate (beginning with 0.5 to 1 mg/kg, increasing 1 mg/kg/week, until 3 to 3.8 mg/kg/day) for those with persistent spasms or EEG alterations after 2 to 4 weeks of VGB treatment. All patients presented seizure cessation; three showed resolution of EEG abnormalities, and three showed normal psychomotor development. 34

A prospective, observational study 35 evaluated VGB as the first treatment drug, including 180 untreated children with IESS starting with 50 mg/kg/day up to 150 mg/kg/day for 14 days. Spasms ceased in 56.1% of the patients after 14 days of treatment, with a mean response time of 5.3 ± 3.02 days. 35 Predictors of response were idiopathic etiology, extensor spasms, and normal psychomotor development before infantile spasms (IS). 35 After 17 years of follow-up, the long-term efficacy with 149 patients resulted in 41.5% of patients showing cessation of IS with VBG and normal neurological status, 57.8% with normal intelligence versus 30.8 and 22.6% in the nonresponders group, respectively ( *p* = 0.001). 35

In a retrospective chart review, 36 68 patients with IESS were treated with VGB starting with 125 to 200 mg/day, weekly increased to 100 mg/kg/day, up to 200 mg/kg/day in case of no response in 2 weeks of treatment. In this population, 56% achieved complete cessation of spasms.

Patients with tuberous sclerosis complex (TSC) showed better control of spasms than patients with spasms from other etiologies. 36 Children with TSC and IESS respond better to VGB than to another antiseizure medication. 37 In a Brazilian case series, 38 five out of seven children with TSC showed cessation of spasms after being treated with VGB. In a retrospective observational study, 39 five patients with TSC and IESS received VGB as first-choice and monotherapy for at least 6 months, beginning in the 1 ^st^ week of seizures and before 12 months of age. All patients with IESS responded to VGB and maintained cessation even after VGB withdrawal. 39 The treated patients did not show severe mental retardation, language deficiency, or autism, compared with five cases of autism and severe mental retardation of the 10 children with TSC who also received VGB but started at least 3 weeks after the onset of seizures. 39

Thirteen children with WS were treated with VGB in monotherapy in a Brazilian child neurology clinic, receiving 96 to 150 mg/kg/day, increased weekly in case of partial response. 40 A total of 54% of children presented complete or at least partial cessation of spasms, and among these patients, 71% showed improvement in cognitive function. 40 In another Brazilian observational case series, 41 with 23 patients using VGB, 69.5% presented complete cessation of spasms, 22% partial response, and 8.5% were nonresponders. The authors used 100 mg/kg/day and increased it to 150 mg/kg/day or combined it with another drug in case of partial response. 41 The drug was suspended in case of no response after 2 weeks and was maintained for 6 months in case of a complete response. 41

A higher dose of VGB is related to higher response and lower relapse rates. An RCT 42 evaluated the efficacy of VGB in IESS treatment, including 221 untreated children to receive either low (18 to 36 mg/kg/day) or high (100 to 148 mg/kg/day) doses. In this study, 15.9% of the patients treated with the high dose presented cessation of spasms for at least 7 consecutive days in the first 14 days of treatment, with no sign of spasms or hypsarrhythmia on EEG, versus 7.0% of the patients treated with the low dose ( *p* = 0.0375). 42 Among those patients, 11.8% receiving the high dose relapsed in a mean time of 162 days, and 25.0% of the low-dose group relapsed in 45 days. 42 In a cohort study 43 with 50 patients with TSC and IESS, the relapse rate among the complete responders to a high dose of VGB (mean dose of 119 mg/kg/day) was 24%, with a median time of 7.4 months.

### Hormonal therapy versus vigabatrin

In the UKISS trial, 10 previously mentioned, 73% of the patients in the hormonal treatment group (70% on prednisolone and 76% on ACTH) presented complete cessation of epileptic spasms after 14 days of treatment, compared with 54% receiving VGB ( *p* = 0.043). Resolution of hypsarrhythmia was also superior with hormonal treatment than with VGB: 81% of patients versus 56%, respectively ( *p* = 0.024). 10 However, prednisolone or ACTH were similar to VGB in seizure control of patients with symptomatic or unknown etiology at the final evaluation at 12 to 14 months 44 : cessation of spasms without relapse occurred in 40% of the hormonal group and in 37% of the VGB group ( *p* = 0.71). Patients with IESS of unknown etiology had superior improvement of mental development with hormonal treatment ( *p* = 0.0003). 44 Results from a long-term evaluation 45 ( *n* = 77), with children at 4 years old, showed that patients with IESS of unknown etiology who received hormonal treatment had superior improvement in communication, living skills, socialization, and motor function than those treated with VGB ( *p* = 0.033).

In a retrospective chart review including 75 children with IESS, 46 hormonal therapy (prednisolone or ACTH) was compared with VGB as initial treatment during an 8-year follow-up. In the group of children treated with prednisolone, 61.1% (95%CI: 38.62–79.69) achieved cessation versus 42.5% (95%CI: 30.33–55.84) with VGB, but without any difference in the relapse rate between treatments (p > 0.05). 46 The response was observed mainly in unknown etiology spasms, where 100% achieved cessation with steroids versus 26.6% with VGB. 46 The mean time to achieve cessation was shorter with hormonal therapy than with VGB (8.18 versus 15.91 days, respectively; *p* = 0.006). 46 A total of 68% of patients used second-line therapy due to no response or relapse of spasms, and 54.6% achieved cessation. 46 Treatment included steroids, VBG, levetiracetam, sodium valproate, or topiramate. 46

A retrospective study 47 ( *n* = 70) evaluated the efficacy of ACTH ( *n* = 16; 1 dose 20 to 40 IU), VGB ( *n* = 5; 35 to 75 mg/kg/day), prednisone ( *n* = 14; 2 to 5 mg/kg/day), valproic acid ( *n* = 17; 15 to 60 mg/kg/day), and nitrazepam ( *n* = 15; 0.5 to 1 mg/kg/day). Patients started with monotherapy for 4 weeks, but in case of failure, clinicians replaced or added another drug. 47 Patients treated with ACTH and those treated with VGB showed the highest response rate: 68.75 and 60% of patients showed complete cessation of spasms, respectively. 47 Adrenocorticotropic hormone was superior to nitrazepam, valproic acid, and prednisone ( *p* < 0.005). 47

In a chart review, 48 38 children with IESS received VGB (12.5 to 25 mg/kg/day, maximum dose 150 mg/kg/day) and 18 received ACTH (40 IU; 2 children received 80 IU); 50% of the patients achieved complete cessation with ACTH in monotherapy versus 55.3% with VGB. 48 For the nonresponders, treatments were switched, and 29.4% presented complete cessation with ACTH and 22.2% with VGB. 48 A total of 55.6% of the patients initially treated with ACTH relapsed, compared with 33.3% with VGB. 48

### Associations

The association of hormonal treatment and VGB has a benefit for a short-term response. In the ICISS study, 49 an open-label RCT including 377 children with IESS, the combination therapy (hormonal with VGB), from day 14 to 42, ceased the spasms in 72% of children versus 57% with hormonal therapy alone ( *p* = 0.002). 49 However, at 18 months, patients from the combined treatment and the hormonal therapy alone did not show any difference in spasms frequency or developmental disabilities. 27 As we have already mentioned in the present paper, it is important to consider the risk of VABAM when associating VGB with ACTH. 31

The association of topiramate with high dose of prednisolone (up to 60 mg/day) does not improve cessation ( *p* = 0.796). 50 Patients not responding to high-dose prednisolone alone adding ACTH therapy also showed limited results, with only 33% of responders. 51

### Experts' opinion

In Brazil, the most used drugs as first-line treatment for IESS are oral prednisolone and VGB. Oral corticosteroids are effective, cheap, and broadly available in our country. Vigabatrin is the first antiepileptic drug to show efficacy through RCTs, but its availability is quite variable in Brazil. It is the first choice for symptomatic IESS, for infants with TSC, and is associated with prednisolone for cases of unknown etiology. Synthetic ACTH (tetracosactide) is part of the first-line treatment of IESS but is not available in public services. There is a whole bureaucratic process to acquire ACTH in Brazil through an importation procedure, resulting in a delay in starting treatment and high costs. Moreover, ACTH requires qualified professionals and a favorable environment for administration (intramuscular route).

## SECOND-LINE THERAPY

### Ketogenic diet

Ketogenic diet (KD) has been used in pharmacoresistant epilepsy 52 and is a therapeutic option for children with IESS not responding to hormonal therapy or VGB. Results of a meta-analysis of nonrandomized clinical trials ( *n* = 345) shows > 50% spasm reduction in 33.62% of the patients after 1 to 6 months of diet. 53

Ketogenic diet compared with high-dose synthetic ACTH (intramuscular, 150 IU/m ^2^ for 2 weeks, then decreasing gradually) shows similar cessation of spasms: 47% with KD and 48% with ACTH. 54 Evaluating patients without previous VGB, 80% treated with ACTH achieve remission versus 47% with KD ( *p* = 0.02). 54 However, the authors suggest caution with interpreting results (underpowered study). 54

Results from a prospective observational study ( *n* = 104) showed 63% of patients with reduction of > 50% in the frequency of spasms with KD after 3 months and of 77% after 24 months. 55

Ketogenic diet used during 8 months, as a third-line therapy, associated with antiseizure medications presents only 18.8% relapse rate after 12 to 39 months, and improvement in the developmental scales. 56 In an RCT, the modified Atkins diet after no response to hormonal therapy, the rate of complete response was 23.9%; 65.2% had > 50% reduction, compared with none of the control group ( *p* ≤ 0.001). 57 In another study, including no responders to hormonal therapy, patients treated with KD had a superior decrease in spasms frequency and remission of hypsarrhythmia than those in the control group ( *p* = 0.025). 58

### Experts' opinion

We recommend the KD for treating IESS after nonresponse to VGB and corticosteroids. Although the ICISS protocol considers KD as a second- or third-line treatment, our opinion is that it should be indicated early for patients considered to be good responders, especially in glut 1 deficiency and TSC. Unfortunately, not all public services in Brazil have nutritionists to implement KD.

### Other antiseizure medications

Antiseizure medications other than VGB show limited response in IESS, and perhaps should not be categorized as second or third choice, but rather former treatment before the strong evidence of hormonal treatment and VGB.

A Brazilian case series 59 reported 13 patients using synthetic ACTH associated with valproate, nitrazepam, or clonazepam for maintenance, whereas 55% relapsed and were treated with VGB. 59

Topiramate is superior to nitrazepam, 60 but monotherapy as first-line treatment is usually not enough and association with ACTH shows better response. 61 Topiramate, as third-line treatment, also has limited results. 62 63

An RCT with levetiracetam shows poor results as second-line treatment, with > 10% of patients responding to the drug. 64 Case reports present limited response, but with up to 71.4% presenting ≥ 50% reduction of spasms. 65 66

#### Experts' opinion

Limited response places these antiseizure medications as second to third-line treatment. Even though oral topiramate, levetiracetam, nitrazepam, clobazam, and valproic acid are easily accessible in Brazil, they are not extensively prescribed for treating IESS because even in developing countries, the therapeutic arsenal for IESS has expanded considerably, with more efficient drugs. Topiramate (up to 15 mg/day) may be associated with VGB treatment or a benzodiazepine. The dose of levetiracetam used is 10 to 20 mg/kg/day, titrating up to 60 mg/kg/day, divided into 2 daily doses, and may be associated with prednisolone and benzodiazepine. Valproic acid is prescribed in a dose of 15 to 20 mg/kg/day, titrating up to 80 mg/kg/day, divided into 2 daily doses, but rarely prescribed in the 1 ^st^ year of life because the risk of fatal hepatotoxicity with valproic acid in this age is high (1/500 in patients from 0 to 2 years old), 67 especially in patients with mitochondrial diseases. 68 Valproic acid may be associated with clobazam, topiramate, and levetiracetam. When prescribing benzodiazepines, we should use 0.5 to 1.0 mg/kg/day of clobazam or nitrazepam and increase the dose until seizures are controlled, or undesirable side effects are presented (bronchorrhea, drooling, excessive sleepiness).

### Surgery

Patients with localized epileptic focus may benefit from surgery for complete control of IESS, 69 especially those refractories to treatment. 69 70 71

Corpus callosotomy is a simple and not expensive neurosurgical procedure, affordable for our healthcare system, and an option for patients without identified lesions on MRI as a second-line treatment for those unresponsive to hormonal and antiseizure medications. 72 In a retrospective chart review of 56 WS cases, 72 42.9% were seizure-free; however, patients presenting psychomotor impairment before the onset of epilepsy have a worse response. 72

Two cases in the literature reported successful treatment with multiple subpial transactions of atypical IESS relapsed to previous therapy with ACTH and VGB, decreasing ≥ 50% of spasms. 73

#### Experts' opinion

Epilepsy surgery may be recommended to selected patients, such as children with a structural lesion, in cases of cortical dysplasia or TSC. If the patient does not respond to first-line treatment and has a focal lesion, surgery should be indicated as soon as possible. Callosotomy may benefit those with intractable IESS and normal neuroimaging not responding to pharmacological therapy. Important to mention that neuroimaging, functional imaging, EEG/VEEG, and electrocorticography play a crucial role in the evaluation of patients with treatment-refractory infantile spasms who have focal cortical damage without evidence of diffuse brain damage, degenerative, or metabolic disease and who may respond to surgery. These exams provide detailed information about the location and nature of epileptiform activity in the brain, helping to guide surgical planning.

## TREATMENT PROTOCOLS

### Screening before starting treatment

The etiology of IESS must be investigated as soon as possible, with patient's history, clinical examination, brain magnetic resonance imaging (MRI), laboratory exams, metabolic workup with a Tandem profile (including lactate and ammonia dosage), and genetic testing with epilepsy panels or exome sequencing. In cases of suspected mitochondrial disease, we should add mitochondrial DNA because seizure, including infantile spasms, is frequently the first symptom of hereditary metabolic disease in children, especially mitochondrial diseases. 74

To confirm the diagnosis of IESS, children must have spasms with or without other types of seizures and an EEG with hypsarrhythmia/multifocal or registered spasms. The spasm must be witnessed by the doctor or by video recording if the EEG is multifocal.

The EEG must be performed at any time of the day, but necessarily in waking (at least 10 minutes after waking up), drowsiness, and sleep (preferably spontaneous, after eating). Initially, the pattern may not be present in its full expression, may be only during sleep, mainly in N1, and may predominate in some regions, especially in posterior areas. As the condition progresses, hypsarrhythmia prevails in wakefulness when it is continuous. Sleep breaks up the hypsarrhythmic pattern and may show multifocal discharges.

To start treatment, hypsarrhythmia or any of its variants is unnecessary, as the spasms may not be accompanied by hypsarrhythmia. Delay therapy because of the absence of hypsarrhythmia is not justified; the presence or absence of hypsarrhythmia on the initial EEG does not change the primary treatment-response outcomes, but the early use of appropriate treatment modifies the outcome of seizure control in patients with and without hypsarrhythmia. 75 After awakening, the electrodecremental response usually starts within 10 minutes, and the spasms have the following characteristics: a diffuse slow wave of high amplitude, brief, fast-paced discharge, and short-term diffuse attenuation.

Patients must not show any signs or symptoms of infection and have normal blood pressure. Laboratory tests include blood count, blood glucose, sodium, potassium, calcium, C-reactive protein, alanine aminotransferase, aspartate aminotransferase, and urine analysis.

### Preparation for the beginning of the protocol

Prophylaric antiparasitc treatment is always necessary before high-dose corticosteroid therapy; albendazole in a single dose of 200 mg for children < 2 years old or mebendazole 100 mg per day for 3 days. 76

Pyridoxine is administered before treatment to rule out a pyridoxine deficiency, not to treat spasms. Intravenous pyridoxine is not available in Brazil, we should use compounded pyridoxine 200 mg per day for 2 to 3 days, intravenously whenever is possible, with concomitant EEG recording. It must not be used if the child only has spasms, but rather if they are associated with other focal seizures without any other cause for the spasms. Children with a defined etiology should not receive pyridoxine.

It is not mandatory to receive the protocol in a hospital environment; clinicians should decide individually and consider the risk of infection. Patients starting ACTH may benefit from hospital admission for early treatment, allowing time to organize subsequent injections. We should consider hospital admission if the social circumstances of the family are complex and if we are uncertain that the treatment will be performed correctly in a domestic environment.

Clinicians should always inform parents of the possible adverse effects of treatment, the importance of hygiene, emphasizing the need to contact them immediately in case of signs or symptoms of an infection, and bring the child for a return visit within 2 weeks.

### Protocols

**Figure 3:**
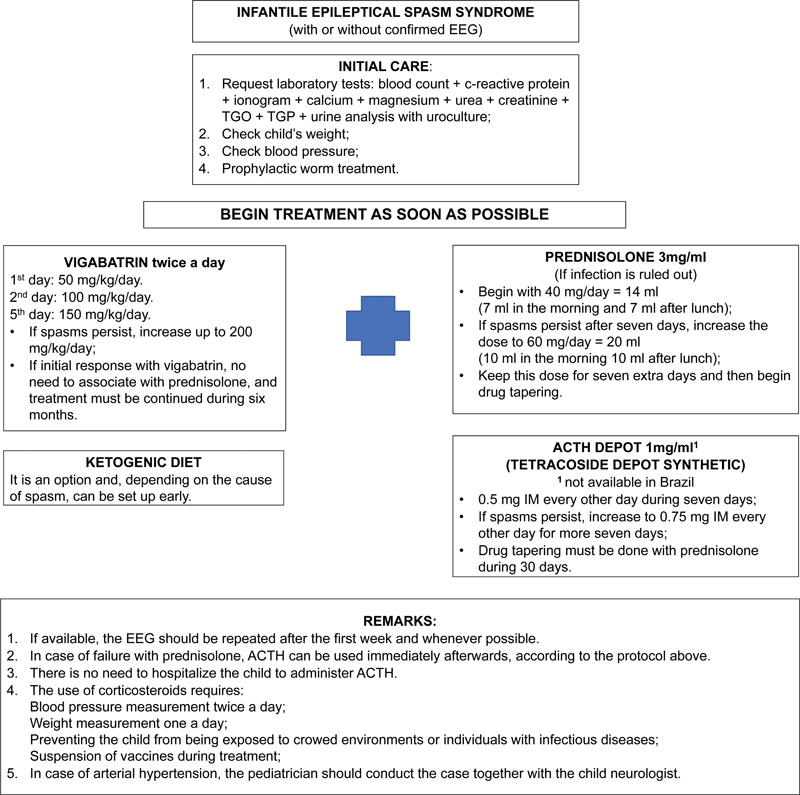
Infantile spasms protocol treatment.

Two well-established protocols are available in the literature and used in several referral centers in Brazil: one from the UKISS study 10 and the other from the ICISS study 49 ( Tables 1 and 2 ). In the UKISS protocol, treatment is based on oral prednisolone, intramuscular ACTH, or VGB ( Table 1 ). In the ICISS, we simultaneously use dual therapy with hormonal therapy (prednisolone or ACTH) and VGB ( Table 2 ). Patients should present cessation of spasms within 14 days. The experts of these consensuses also provide a protocol in the present paper, more adapted to the health resources in Brazil, as shown in Fig. 3 .

**Table 1:** UKISS protocol: monotherapy with prednisolone or ACTH or vigabatrin

	Prednisolone	ACTH	Vigabatrin
**Presentation**	Oral 3 mg/ml	Intramuscular Synacthen Depot 1 mg/ml	Oral Sabril 500 mg
**Initial dose**	10 mg 4 times a day (40 mg/day)	0.5 mg (40 IU) every other day	50 mg/kg/day divided in 2 times/day for 1 day
**Drug titration**	After 1 week: if spasms persist, 20 mg thrice a day (60 mg/day)	After 1 week: if spasms persist, 0.75 mg (60 IU) every other day	100 mg/kg/day divided in 2 times/day for 72 hours After 5 days, if spasms persist, 150 mg/kg/day divided in 2 times/day
**Drug tapering**	After 2 weeks If 40 mg/day: decrease 10 mg each 5 days. If 60 mg/day: decrease to 40 mg/day within 5 days, then decrease 10 mg each 5 days	After 2 weeks If 40 IU, use oral prednisolone 30 mg/day divided in 3 to 4 times/day, decrease 10 mg each 5 days. If 60 IU, use oral prednisolone 40 mg/day divided in 3 to 4 times/day, decrease 10 mg each 5 days	Maintenance for 6 months

Abbreviation: ACTH, adrenocorticotropic hormone.

**Table 2:** ICISS protocol: Vigabatrin associated with prednisolone or ACTH

	Vigabatrin	Prednisolone	ACTH
**Presentation**	Oral Sabril 500 mg	Oral 3 mg/ml	Intramuscular Synacthen Depot 1 mg/ml
**Initial dose**	50 mg/kg/day divided in 2 times/day for 1 day	10 mg 4 times a day (40 mg/day) for 2 weeks	0.5 mg (40 IU) every other day
**Drug titration**	100 mg/kg/day divided in 2 times/day during 72 hours After 5 days if spasms persist, 150 mg/kg/day divided in 2 times a day	If spasms persist after 1 week or relapse after 8 to 14 days: 20 mg thrice a day (60 mg/day)	If spasms persist after 1 week or relapse after 8 to 14 days: 0.75 mg (60 IU) every other day
**Drug tapering**	Use for 3 months, then reduce in 4 weeks	After 2 weeks If 40 mg/day: decrease 10 mg each 5 days. If 60 mg/day: 40 mg/day for 5 days, then 20 mg/day for 5 days, then 10 mg each 5 days	After 2 weeks If 40 IU, use oral prednisolone 30 mg/day divided in 3 to 4 times/day, decrease 10 mg each 5 days. If 60 IU, use oral prednisolone 40 mg/day divided in 3 to 4 times/day, decrease 10 mg each 5 days

Abbreviation: ACTH, adrenocorticotropic hormone.

It is up to the treating physician to decide which protocol is more feasible and appropriate for their patient, facing the limitations of our health system. Some departments in Brazil initially use intramuscular ACTH or oral prednisone in the inability to acquire ACTH, then VGB as a second-line treatment. Another option is to start with VGB alone, especially when patients present an altered neurological examination and/or neuroimaging, that is, symptomatic IESS, followed by ACTH or high-doses prednisolone in case of refractoriness.

It is important to mention that in patients with IESS and TSC, VGB is the first-line treatment, as it is the most effective treatment in this population. Adrenocorticotropic hormone should be considered as adjunctive treatment or an alternative to VGB. In spasms refractory to this first-line treatment, the KD may be indicated, as the response to treatment is good, and these patients should be referred for evaluation of surgical possibility. 77

The ACTH available in Brazil is the synthetic tetracosactide depot (Synacthen Depot, 1 mg/ml, Novartis). Adrenocorticotropic hormone should be administered intramuscularly, in the morning, each other day.

We should prescribe gastric protection for patients using corticosteroids, with omeprazole 1 mg/kg/day in the morning on an empty stomach.

We should carry out weekly laboratory tests (blood count, blood glucose, sodium, potassium, calcium, alanine aminotransferase, aspartate aminotransferase, urine analysis) and chest X-ray. An EEG is performed after the 1 ^st^ week if the spasms ceased, then on the 14 ^th^ and on the 42 ^nd^ day (6 weeks of treatment). The 12 to 24-hour video-EEG should be recommended to certify the absence of spasms or periods of hypsarrhythmia.

In conclusion, IESS is the most common epileptic and developmental encephalopathy in the 1 ^st^ year of life, with a poor prognosis. Therefore, early and aggressive treatment is important, even in infants who do not meet the diagnostic criteria for WS (infantile spasms + hypsarrhythmia + involution or developmental arrest). Treatment of IESS is challenging, especially in countries with limited access to healthcare. In this consensus, we gathered the best evidence found in the medical literature and the available resources in our country to provide optimal management of IESS in Brazil.
